# A Chemical Method for Labeling Lysine Methyltransferase Substrates

**DOI:** 10.1002/cbic.201000433

**Published:** 2010-11-17

**Authors:** Olivier Binda, Michael Boyce, Jason S Rush, Krishnan K Palaniappan, Carolyn R Bertozzi, Or Gozani

**Affiliations:** a Department of Biology, Stanford UniversityStanford, CA 94305-5020 (USA), Fax: (+1) 650-725-8309 E-mail: ogozani@stanford.edu; c Department of Chemistry, University of CaliforniaBerkeley, CA 94720 (USA); d Department Molecular and Cellular Biology, University of CaliforniaBerkeley, CA 94720 (USA); e Howard Hughes Medical Institute, University of CaliforniaBerkeley, CA 94720 (USA)

**Keywords:** click chemistry, cycloaddition, epigenetics, lysine methyltransferases, SETDB1

## Abstract

Several protein lysine methyltransferases (PKMTs) modify histones to regulate chromatin-dependent cellular processes, such as transcription, DNA replication and DNA damage repair. PKMTs are likely to have many additional substrates in addition to histones, but relatively few nonhistone substrates have been characterized, and the substrate specificity for many PKMTs has yet to be defined. Thus, new unbiased methods are needed to find PKMT substrates. Here, we describe a chemical biology approach for unbiased, proteome-wide identification of novel PKMT substrates. Our strategy makes use of an alkyne-bearing *S*-adenosylmethionine (SAM) analogue, which is accepted by the PKMT, SETDB1, as a cofactor, resulting in the enzymatic attachment of a terminal alkyne to its substrate. Such labeled proteins can then be treated with azide-functionalized probes to ligate affinity handles or fluorophores to the PKMT substrates. As a proof-of-concept, we have used SETDB1 to transfer the alkyne moiety from the SAM analogue onto a recombinant histone H3 substrate. We anticipate that this chemical method will find broad use in epigenetics to enable unbiased searches for new PKMT substrates by using recombinant enzymes and unnatural SAM cofactors to label and purify many substrates simultaneously from complex organelle or cell extracts.

## Introduction

The latest human genome annotation predicts 52 protein lysine methyltransferases (PKMTs) and 33 protein lysine demethylases (PKDMTs) based on sequence similarities to known catalytic domains. The number of enzymes involved in the addition and removal of methyl moieties on lysine residues suggests that methylation is dynamic and highly regulated, and numerous undiscovered substrates could exist. Moreover, several of these enzymes themselves are uncharacterized or poorly studied. Thus, important questions regarding the biological relevance and biochemical properties of these enzymes remain unanswered. In addition, recent reports have linked PKMTs with the etiology of human diseases, such as cancer,^1^ Huntington’s disease,^2^ immunodeficiency syndromes,^3^ and growth defects.^4^ Importantly, several PKMTs methylate nonhistone substrates,^5^ such as the tumor suppressor p53,^6^ the estrogen receptor ERα,^7^ the heterochromatin protein HP1α,^8^ the DNA methyltransferase DNMT1,^9^ the ATPase Reptin,^10^ and others. Thus, extensive proteomic profiling of PKMT substrates will be critical for identifying new nonhistone substrates and novel histone substrate sites of PKMTs, and for understanding the biological roles and pathological implications of lysine methylation.

SETDB1 is a histone H3 lysine 9 methyltransferase^11^ that catalyses lysine mono-, di- and trimethylation.^12^ Recent work has identified a critical role for SETDB1 in embryonic stem (ES) cell differentiation^13^ and proviral silencing in ES cells,^14^ as well as a central function in transcriptional silencing as part of a major repressive complex.^15^ Here, we use SETDB1 as a model enzyme to develop a chemical biology method to identify novel PKMT substrates.

**Scheme 1:**
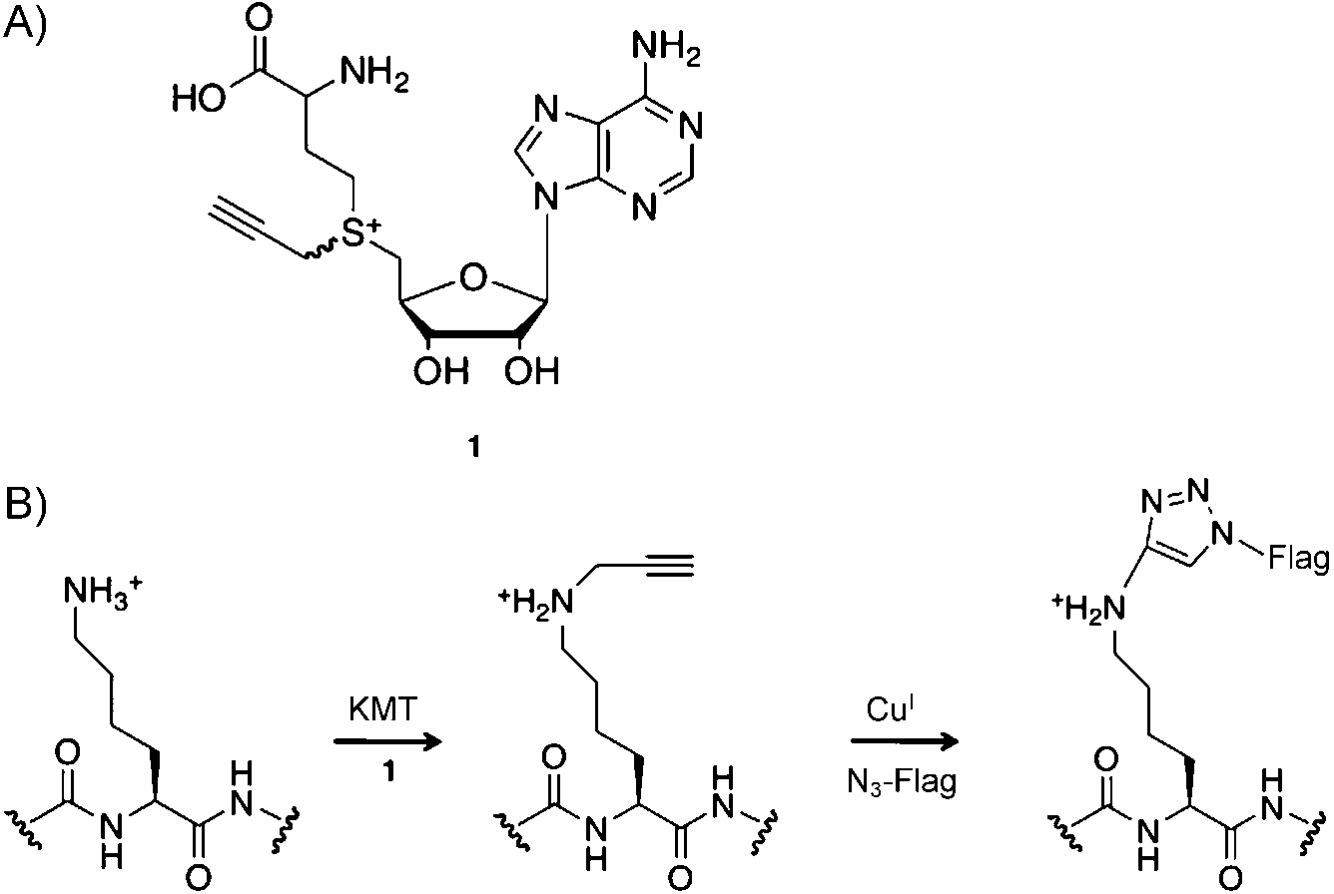
A chemical biology method for labeling lysine methyltransferase substrates. A) Alkyne–SAM analogue **1**. B) PKMT substrate labeling method: enzymatic transfer by a PKMT of a terminal alkyne from **1** to a protein substrate is followed by CuAAC to ligate an azido-FLAG epitope to the substrate protein.

Lysine methylation events have historically been identified by candidate approaches, limiting the discovery of unanticipated but biologically relevant substrates. Therefore, unbiased methods for labeling and characterizing PKMT substrates would be of great utility. To address this need, we have developed a chemical biology approach based on copper-catalyzed azide-alkyne cycloaddition (CuAAC) chemistry. CuAAC methods have found broad use in studying many types of cellular processes, including such post-translational protein modifications as glycosylation,^16^ lipidation,^17^ and acetylation,^18^ as well as DNA replication^19^ and RNA dynamics.^20^ Because neither alkynes nor azides appreciably react with any functional groups found in natural biomolecules, CuAAC exhibits exquisite specificity and very low background reactivity in biological samples, making it an ideal approach for identification of novel PKMT substrates. Here, we describe the synthesis of an alkyne-functionalized *S*-adenosylmethionine (SAM) analogue (Scheme 1 A, **1**), and report that it is accepted, in vitro, by SETDB1, which transfers the alkyne onto a recombinant histone H3 substrate. The resulting alkyne-tagged lysine moiety can then be treated with azide-bearing reporters, such as a FLAG epitope, by CuAAC (Scheme 1 B). The modified substrates, labeled with recombinant PKMTs in such complex mixtures as organelle or cell extracts, could then be purified by anti-FLAG affinity chromatography and identified by mass spectrometry. We expect that the alkyne-bearing SAM analogue approach will allow the use of chemical methods to investigate protein methylation in a variety of experimental contexts and advance the proteomic identification of methylated proteins.

## Results and Discussion

### Alkyne–SAM synthesis, purification, and analysis

Previous work has shown that synthetic SAM analogues can serve as cofactors for other classes of methyltransferases, including DNA methyltransferases.^21^ Specifically, analogues with a double or triple bond β to the sulfonium center of the cofactor allow for the efficient enzymatic transfer of extended groups onto DNA, probably because of the conjugative stabilization of the p orbital of the reactive carbon, which compensates (at least in part) for the steric hindrance imposed by the larger synthetic cofactor.^21^ Based on this work, we reasoned that **1**, a synthetic SAM analogue with a propargyl-substituted sulfonium functionality, might be accepted by PKMTs. Importantly, the use of **1** as a cofactor by a PKMT would result in the transfer of a terminal alkyne moiety onto the PKMT substrate and allow for selective probe conjugation by CuAAC.

During synthesis and HPLC purification we observed one major chromatographic peak with associated mass spectra containing the predicted product mass. NMR analysis indicated that both diastereomers of **1** were formed, as predicted, and were present in an approximate 1.67:1 ratio, although initial attempts to separate the diastereomers chromatographically have been unsuccessful. Nevertheless, as other unnatural SAM analogues have been used successfully in biological assays as mixtures of diastereomers,^22^ we evaluated **1** in our PKMT assays.

### Lysine methyltransferase reaction with alkyne–SAM

**Figure 1:**
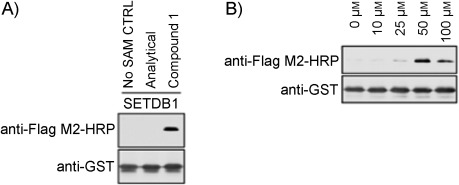
Alkyne–SAM assessment in lysine methylation assays. A) SETDB1 was incubated with GST–H3_tail_ and **1** or controls (no cofactor, trace material from analytical HPLC prep). Following the methyltransferase reaction, the product was treated with the azide-FLAG and the samples were analyzed by SDS-PAGE and immunoblotting by using the indicated antibodies. B) Methyltransferase reactions were performed as in panel A, but by using **1** in increasing amounts.

We tested **1** in an in vitro lysine methylation assay with recombinant SETDB1 and recombinant histone H3 tail encompassing amino acids 1–42 of H3 fused to GST (GST-tagged H3_tail_). Then, following GST purification of GST–H3_tail_, CuAAC was performed by using an azide-FLAG probe. As shown in Figure 1 A, SETDB1 efficiently transferred the alkyne group from **1** to GST–H3_tail_, as detected by anti-FLAG immunoblotting. Anti-GST immunoblotting confirmed that equivalent amounts of GST–H3_tail_ were used in this assay (Figure 1 A, lower panel). We then optimized the amount of **1** for the most effective modification conditions. Using varying amounts of **1**, ranging from 0 to 100 μm, we found that the optimal concentration of **1** for H3 modification by SETDB1 is 50 μm (Figure 1 B). As a loading control, anti-GST immunoblotting was performed to confirm equal level of substrate in each PKMT assay reaction. Typically, natural SAM is used at concentrations in the lower micromolar range (3 to 160 μm) in similar assays,^23^ in agreement with our results with **1**. However, we note that our experiment probably overestimates the concentration of analogue needed for optimal activity, as it is likely that only one diastereomer is accepted by SETDB1, as with natural SAM.

### Detection and FLAG purification of the substrate

**Figure 2:**
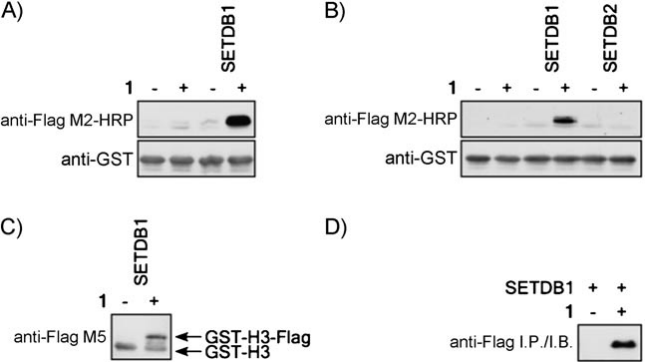
Alkyne–SAM transfer onto histone H3 by SETDB1. A) Lysine methylation assays performed in the absence or presence of **1** and SETDB1. B) Lysine methylation assays conducted with SETDB2 along with SETDB1 as a control. C) Lysine methylation assays with **1** analyzed with the M5 anti-FLAG antibody to show the differential migration of the alkyne-modified substrate. D) Recombinant histone H3 was immunoprecipitated by using FLAG M2–agarose after CuAAC.

After optimization of PKMT assay conditions with **1**, we validated SETDB1 activity using 50 μm of **1**. Lysine methylation reactions were conducted in the absence or presence of either SETDB1 or **1**. Figure 2 A shows that the anti-FLAG antibody detects the GST–H3_tail_ solely in the presence of SETDB1 and **1**, demonstrating the transfer of the alkyne moiety onto the substrate. In contrast, SETDB2, a putative PKMT highly homologous to SETDB1, did not transfer the alkyne moiety from **1**; this suggests that **1** might provide stringent selectivity among PKMTs (Figure 2 B).

Ligation of the FLAG epitope (DYKDDDDK; ∼1012 Da) through CuAAC should alter the migration of the histone substrate relative to the unmodified form. To test this, we used the anti-FLAG M5 monoclonal antibody, which, due to the relatively high amount of GST–H3_tail_, also nonspecifically recognizes the unlabeled substrate. Figure 2 C shows that the modified H3 migrates on SDS-PAGE slightly slower than the unmodified form, as predicted.

To further validate that the FLAG epitope added to the PKMT substrate by click chemistry is indeed functional, we conducted an immunoprecipitation experiment on SETDB1 GST–H3_tail_ PKMT reactions in the absence or presence of **1**. Figure 2 D shows that recombinant H3 was pulled-down by the anti-FLAG antibody only when the methylation reaction was carried out in the presence of **1**. Thus, alkyne modified substrates can be purified with antibodies; this suggests that novel substrates could be purified in this manner and analyzed by mass spectrometry.

Recently, a chemical biology approach was used to identify novel lysine acetyltransferase substrates.^18^ Yang et al. synthesized a series of acetyl-CoA analogues with alkyne moieties of variable length in experiments with the histone acetyltransferase p300.^18^ Interestingly, p300 could not transfer the alkyne moiety from the butynoyl-CoA, but successfully achieved the transfer from pentynoyl-CoA and, to a certain extent, from hexynoyl-CoA.^18^ By analogy, these results suggest that further optimization might yield additional future alkyne–SAM analogues that could be useful either for specific PKMTs or several PKMTs. Indeed, a very recent report indicates that this is the case for specific fungal and human PKMTs that use a SAM analogue distinct from the one we report here.22b In addition, Osborne et al. synthesized an *N*-mustard SAM derivative, which is transferred to arginine by the protein arginine methyltransferase, PRMT1, and proposed that alkyne–SAM could be used for proteomic identification of novel PRMT substrates.^24^ Interestingly, the *N*-mustard SAM derivative was accepted as a cofactor by rebeccamycin methyltransferase^25^ and DNA methyltransferases^26^ to modify their respective substrates. Thus, alkyne–SAM analogues have the potential to facilitate the proteomic identification of novel substrates for various families of methyltransferases, and possibly other applications, such as methyl-CpG genomic DNA and mRNA 7-methylguanosine cap tagging. However, we found that other methyltransferases, including SET7, SMYD2, PRMT1, CARM1, and PRDM8, -10, and -16, were unable to accept **1** in in vitro assays, as SETDB1 does (data not shown). These results suggest that **1** might provide a relatively specific reagent for the study of SETDB1, and perhaps other closely related PKMTs, the substrates and functions of which remain poorly understood.

## Conclusions

We note that the work described here provides a “jumping-off” point for the design and synthesis of future generations of alkyne- and azide-functionalized SAM analogues with improved properties, including cell permeability and acceptance by a wider range of PKMTs.

## Experimental Section

**Alkyne–SAM synthesis and purification:** Alkyne–SAM analogue **1** was synthesized essentially according to the method of Dalhoff et al.22a Briefly, *S*-adenosyl-L-homocysteine (50 mg, 0.13 mmol) was dissolved in a 1:1 mixture of formic acid and acetic acid (7.5 mL) on an ice bath. Propargyl bromide (1.2 mL, 7.8 mmol) was added slowly over 5 min, the reaction was allowed to warm to room temperature and stirred for 4 days. The reaction was then diluted with water (75 mL) and washed three times with diethyl ether (12.5 mL each). The aqueous layer was frozen and lyophilized. Lyophilized material was dissolved in water with TFA (0.1 %) and purified by RP-HPLC by using a Rainin Instruments Dynamax SD-200 system equipped with a Varian UV/vis detector (model 345) and a Microsorb C18 analytical column (4.6×250 mm) with a flow rate of 1 mL min^−1^ or a preparative column (21.4×250 mm) with a flow rate of 20 mL min^−1^. HPLC samples were filtered with a Pall Life Sciences Acrodisc CR 13 mm syringe filter equipped with a 0.2 μm PTFE membrane prior to injection. The product was purified with an isocratic elution of TFA (0.1 %) in water; *t*_R_ (**1**)=9.1 min. ^1^H NMR (500 MHz, D_2_O): *δ*=8.434 (s, 1 H; H_A_), 8.430 (s, 1 H; H_B_), 8.43–8.41 (m, 1.2 H; H_A_/H_B_), 6.15 (d, *J*=3.0 Hz, 1 H; H_A_), 6.12 (d, *J*=4.0 Hz, 0.6 H; H_B_), 4.88–4.83 (m, 1.4 H; H_A_/H_B_), 4.64 (dd, *J*=6.5, 5.5 Hz, 1 H; H_A_), 4.61 (dd, *J*=6.0, 5.5 Hz, 0.6 H; H_B_), 4.55 (ddd, *J*=7.0, 6.0, 4.0 Hz, 1.8 H; H_A_/H_B_), 4.44 (d, *J*=2.5 Hz, 0.9 H; H_B_), 4.35 (ddd, *J*=17.5, 12.5, 2.5 Hz, 2 H; H_A_), 4.09 (dd, *J*=13.0, 3.0 Hz, 1 H; H_A_), 4.05–4.01 (m, 1.5 H; H_B_), 3.95 (d, *J*=2.5 Hz, 1 H; H_A_), 3.94 (s, 1 H; H_A_), 3.73–3.58 (m, 3 H; H_A_/H_B_), 3.24 (t, *J*=2.5 Hz, 0.4 H; H_B_), 3.12 (t, *J*=2.5 Hz, 1 H; H_A_), 2.47–2.29 (m, 3.6 H; H_A_/H_B_); ^13^C NMR (150 MHz, D_2_O, only major diastereomer, A, reported): *δ*=170.6, 162.9, 162.7, 144.5, 143.2, 117.3, 115.3, 90.0, 81.3, 79.1, 73.2, 73.1, 51.3, 41.8, 36.1, 30.2, 24.5; ESI-HRMS calcd for C_17_H_23_N_6_O_5_S^+^: 423.1445 [*M*]^+^, found 423.1447.

**Plasmids, cDNA, and antibodies:** The histone H3 tail was inserted in-frame upstream of GST in pGEX (Pharmacia). SETDB1 cDNA was HA-tagged and inserted in pcDNA3 (Invitrogen). The antibodies used were anti-GST–HRP (ab3416, Abcam) and anti-FLAG (M2–HRP and M5–HRP, Sigma).

**Recombinant protein purification:** Briefly, *E. coli* strain BL21 DE3 (Stratagene) was transformed with appropriate pGEX plasmids, and protein expression was induced and purified, as described previously.^27^

**Lysine methylation assay:** The KMT assays were essentially performed as described previously,^28^ but **1** was used instead of ^3^H-*S*-adenosylmethionine. The reactions were incubated for 2–4 h at 37 °C.

**Click reaction:** The samples were cleared of **1** by either GST-pull-down or TCA precipitation. Then, the protein samples were incubated in the presence of sodium ascorbate (1.5 mm), azido-FLAG (100 μm), Tris[(1-benzyl-1*H*-1,2,3-triazol-4-yl)methyl]amine (100 μm), and CuSO_4_ (1 mm) at room temperature for 60 min. Reactions were stopped by addition of Laemmli buffer and analyzed by immunoblot.
